# Non-invasive Ventilation in Adult Cancer Patients With Acute Respiratory Failure: A Systematic Review of Clinical Outcomes and Predictors of Failure

**DOI:** 10.7759/cureus.98833

**Published:** 2025-12-09

**Authors:** Mohamad Abu Zaher, Osman El Jundi, Hanan Arain

**Affiliations:** 1 Renal, Broomfield Hospital, Chelmsford, GBR; 2 Respiratory Medicine, Istanbul Atlas University, Istanbul, TUR; 3 Respiratory Medicine, Anglia Ruskin University, Chelmsford, GBR

**Keywords:** acute respiratory failure (arf), bilevel positive airway pressure-bipap, continuous positive airway pressure (cpap), haemato-oncology, high-flow nasal cannula (hfnc), intensive respiratory care, niv (non-invasive ventilation)

## Abstract

Acute respiratory failure (ARF) is a leading cause of unexpected admissions to the ICU in adults with active solid or haematologic cancer. Early trials highlighted some benefits of non-invasive ventilation (NIV) delivered with supplemental oxygen versus conventional oxygen. Whether this is still maintained in the high-flow nasal cannula (HFNC) era is doubtful. Therefore, a Preferred Reporting Items for Systematic Reviews and Meta-Analyses (PRISMA)-guided, scenario-based systematic review was conducted to evaluate the effectiveness and harms of NIV versus oxygen/HFNC and to identify predictors and outcomes of NIV failure in adult cancer patients with ARF. Searches on PubMed from inception to 31 August 2025, Scopus, and the Cochrane Library from 2010 to 31 August 2025 were completed. In this review of trials and studies, primary outcomes were NIV failure, defined as intubation during the index episode, and in-hospital mortality. Secondary outcomes were length of stay, complications, and predictors of NIV failure. Two reviewers screened, extracted, and assessed risk of bias using Cochrane Risk of Bias 2 (RoB 2) and the Newcastle-Ottawa Scale. We did not perform a formal meta-analysis, and studies were compared qualitatively. Twenty-six studies met the inclusion criteria, including four randomised trials and 22 cohorts, with a pooled sample of approximately 12,000 patients. In de novo hypoxemic respiratory failure, the largest recent trial and adjusted cohorts showed no decline in intubation or mortality rates with early NIV versus oxygen or high-flow nasal cannula. In cancer-related acute respiratory distress syndrome (ARDS), a form of ARF, NIV failure was common at about 60% to 80% and greatly linked to death. In the case of NIV failure, independent predictors of failure included shock, lower arterial oxygen partial pressure to inspired oxygen fraction ratio (PaO₂/FiO₂), invasive fungal infection, higher severity scores, and undetermined aetiology. However, in cases of cancer and acute respiratory failure with cardiac dysfunction, observational data suggested lower ICU mortality with early NIV. Limitations of this review include heterogeneity, residual confounding, and variable protocols. To conclude, benefits of NIV in cancer-related ARF are context dependent. There is no clear advantage of NIV over HFNC/oxygen in de novo hypoxemia. In addition, high failure risk in ARDS with NIV mandates vigilant escalation. A context-specific NIV approach rather than a blanket one is highly recommended in the HFNC era.

## Introduction and background

Acute respiratory failure is a prime reason for unplanned ICU admission in adults with active solid tumours or haematologic malignancies. In these cases, short-term mortality is high, and it is even higher when acute respiratory distress syndrome is present [1,2]. In immunocompromised patients with hypoxemic respiratory failure, especially when the cause is uncertain or an opportunistic infection is suspected, both intubation and death remain prevalent [3,4]. Non-invasive ventilation (NIV) delivered as non-invasive positive pressure ventilation with supplemental oxygen via mask is attractive because it can improve oxygenation and reduce the work of breathing, while avoiding some of the harms of invasive ventilation. Early single-centre studies in immunosuppressed patients, including the trial by Hilbert and colleagues, reported lower intubation and mortality rates with early NIV compared to conventional oxygen. This finding helped drive the acceptance of NIV in oncology critical care [5].

Today the care outlook is different. High-flow nasal cannula oxygen therapy is extensively accessible, and supportive care pathways are better standardised. This progress sheds a shadow of uncertainty on the added value of NIV. This is most significant in de novo hypoxemic respiratory failure, that is not due to chronic obstructive pulmonary disease (COPD) exacerbation or acute cardiogenic pulmonary oedema [6,7]. Clinicians need to know which presentations and settings benefit the most from NIV, which are better treated by high-flow nasal cannula, and when persistent application of NIV may delay necessary intubations.

A scenario-based systematic review of adults with cancer and acute respiratory failure was performed for the purpose of this study. We examined five questions: first, the effectiveness of NIV versus oxygen or high-flow nasal cannula in de novo hypoxaemia; second, failure rates and outcomes in established acute respiratory distress syndrome; third, risk factors of NIV failure; fourth, prognosis after NIV failure; and fifth, ward or pre-ICU use of NIV in selected haematology populations with, or at high risk of, acute respiratory failure.

## Review

Methods

Study Design and Framework

This systematic review followed Preferred Reporting Items for Systematic Reviews and Meta-Analyses (PRISMA) 2020 guidelines under a pre-specified protocol registered in the International Prospective Register of Systematic Reviews (PROSPERO; CRD420251102556). The aim was to evaluate the effectiveness and harms of non-invasive ventilation in adults with active cancer and acute respiratory failure, and to identify predictors of NIV failure. 

Population, Intervention, Comparator, Outcome (PICO)

Population: Adults aged 18 years or older with active solid or haematologic malignancy and acute respiratory failure in hospital, ICU, haematology, or haematopoietic stem cell transplantation wards.

Intervention: NIV delivered as continuous positive airway pressure (CPAP) or bilevel positive airway pressure (BiPAP) via mask or helmet.

Comparators: Conventional oxygen, high-flow nasal cannula, invasive mechanical ventilation, or none.

Outcomes: Intubation or NIV failure, mortality with in-hospital as the primary time point, ICU or time-bound mortality when available, length of stay, complications, and predictors of NIV failure.

Search Strategy

We searched PubMed from inception to 31 August 2025 (no date limits) and Scopus and Cochrane CENTRAL from 2010 to 31 August 2025. Pre-2010 studies were therefore eligible if identified via PubMed. Searches were limited to English. Concepts combined cancer or neoplasms, acute respiratory failure or ARDS, and non-invasive ventilation including CPAP and BiPAP. Reference lists of included studies and relevant reviews were screened. Full database-specific search strings for PubMed, Scopus, and Cochrane CENTRAL are provided in Appendix 1 (Appendix 1a-1c).

Eligibility Criteria

Inclusion: Human studies in English that were randomised trials, cohort studies, or case series with at least five cancer patients treated with NIV for acute respiratory failure, and that reported at least one prespecified outcome.

Exclusion: Paediatric populations, animal studies, case reports, reviews or editorials, studies without outcome data, studies focused only on invasive ventilation, and prophylactic postoperative or post-extubation NIV when acute respiratory failure was not present. Postoperative acute respiratory failure treated with NIV was eligible when the study explicitly defined acute respiratory failure.

Study Selection and Data Extraction

Two reviewers independently screened titles and abstracts and then full texts. Disagreements were resolved by discussion. Data was extracted with a piloted form into a prespecified spreadsheet. We recorded study characteristics, setting, design, sample size, cancer type and status, immunosuppression where reported, acute respiratory failure phenotype and aetiology, details of the intervention and comparator, and outcomes including intubation or NIV failure, mortality, length of stay, and complications. Do-not-intubate status was extracted where available. For predictors we extracted variables entered in multivariable models, adjusted effect sizes with 95% confidence intervals, model type, and covariates.

Risk of Bias Assessment

Two reviewers assessed risk of bias by design using Cochrane Risk of Bias 2 (RoB 2) [8] for randomised trials, and the Newcastle-Ottawa Scale [9]. Discrepancies were resolved by common consent.

Data Synthesis and Analysis

We performed a narrative qualitative synthesis only. We prespecified no meta-analysis because of heterogeneity in populations, acute respiratory failure definitions, NIV strategies and interfaces, and outcome time points. Where scenario-level summaries were presented (e.g., median NIV failure and mortality), these were calculated as simple medians with interquartile ranges across contributing studies. Results are organised by scenario: de novo hypoxaemia, acute respiratory distress syndrome (ARDS), ward or pre-ICU use, and outcomes after NIV failure. For predictors, we reported adjusted associations, noted how consistent they were across studies, and mentioned any thresholds that were reported. Univariate results were treated as hypothesis-generating.

Results

Study Selection and Characteristics

**Figure 1:**
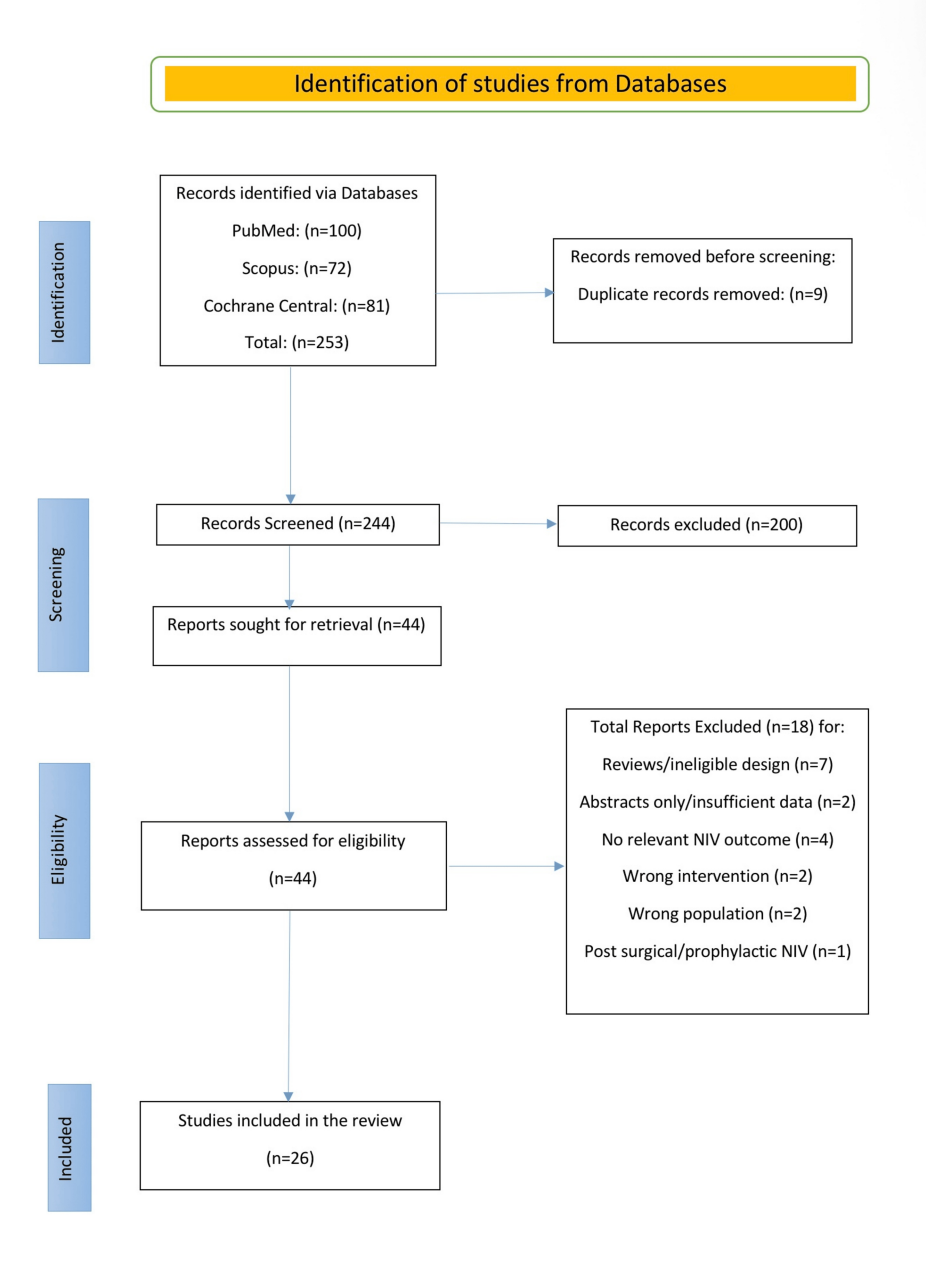
PRISMA 2020 study selection for NIV in cancer-related acute respiratory failure Records were identified from PubMed (n = 100), Scopus (n = 72), and Cochrane CENTRAL (n = 81), yielding 253 records. After removal of nine duplicate records, 244 records were screened by title and abstract, and 200 were excluded. Forty-four reports were sought and assessed for eligibility; 18 were excluded with the following reasons: reviews/ineligible design (n = 7), abstracts only/insufficient data (n = 2), no relevant NIV outcome (n = 4), wrong intervention (n = 2), wrong population (n = 2), and post-surgical/prophylactic NIV (n = 1). Twenty-six studies were included in the qualitative synthesis. PRISMA = Preferred Reporting Items for Systematic Reviews and Meta-Analyses, NIV = non-invasive ventilation.

Twenty-six studies (four randomised controlled trials (RCTs), 22 cohorts) were included. The PRISMA flow chart is shown in Figure 1. Across studies, around 12,000 adult cancer patients with acute respiratory failure (ARF) received non-invasive ventilation (NIV). Populations were predominantly suffering from haematologic malignancies (acute leukaemia, lymphoma, myeloma), with additional solid-tumour cohorts. ARF aetiologies were most often infection (bacterial/fungal pneumonia, sepsis), followed by pulmonary oedema, tumour infiltration, or undetermined cause. NIV was commonly used as first-line ICU support via oronasal mask in pressure-support mode and in all included studies it was delivered with supplemental oxygen. Unless declared, “NIV failure” indicates intubation during the index ARF episode and mortality refers to in-hospital mortality. Per-study characteristics and outcomes are shown in Table 1.

**Table 1:** Characteristics of included studies evaluating non-invasive ventilation in adults with cancer and acute respiratory failure Study-level details include first author/year, country and setting (ICU/ward/HSCT), design (RCT/cohort), sample size, cancer type (solid/haematologic), ARF aetiology (infection, pulmonary oedema, tumour infiltration, undetermined), severity indices (SAPS II/SOFA/APACHE II), respiratory support strategy (NIV mode/interface: CPAP or bilevel, mask/helmet), comparator (oxygen or HFNC, invasive ventilation when applicable), predefined NIV stop rules (yes/no), do-not-intubate (DNI) proportion, primary outcomes (NIV failure = intubation during index ARF episode, in-hospital or ICU mortality), and secondary outcomes (length of stay, complications). Values are reported as n/N (%), mean ± SD, or median (IQR) as provided in the source article. Where multicentre datasets potentially overlap, the larger or most recent cohort is flagged in footnotes; totals should be interpreted as approximate rather than unique patient counts. Risk of bias was assessed with Cochrane Risk of Bias 2 (RoB 2) [8] (trials) and Newcastle-Ottawa Scale [9] (cohorts). HM = haematologic malignancy, HSCT = haematopoietic stem cell transplantation, ARF = acute respiratory failure, ARDS = acute respiratory distress syndrome, HFNC = high-flow nasal cannula, IMV = invasive mechanical ventilation, NIV = non-invasive ventilation, CPAP = continuous positive airway pressure, LOS = length of stay, RCT = randomised controlled trial, ITU = intensive therapy unit (ICU), NPPV = non-invasive positive-pressure ventilation, SAPS II = Simplified Acute Physiology Score II, SOFA = Sequential Organ Failure Assessment, APACHE II – Acute Physiology and Chronic Health Evaluation II.

Study	Year	Country	Design	Setting	ARF phenotype	N (NIV)	Population (HM vs solid)	Contrast type	DNI included?	NIV approach (mode/interface; timing)
Liu et al. [1]	2017	Canada	Retrospective cohort	ICU	ARDS	79	Haematologic	Single-arm NIV cohort	Included	First line; mask; median, 41 minutes after arrival to ITU
Bris et al. [2]	2024	France	Retrospective cohort	ICU	ARDS	1,373	Mixed	Descriptive ARDS cohort (national; NIV subgroup)	Not specified	Non-invasive ventilation applied in 1,373 cancer patients. Within index ICU admission; exact timing not captured
Contejean et al. [3]	2016	France and Belgium	Post hoc analysis of a prospective multicentre cohort	ICU	De novo hypoxemia	205	Haematologic	ARF aetiology, determined vs undetermined	Not specified	Started in 205/604 patients (33.9%) at (or within hours of) ICU admission
Azoulay et al. [4]	2017	Multinational	Prospective cohort	ICU	De novo hypoxemia	232	Mixed	NIV vs HFNC	Not specified	Initiated in 232/915; at ICU admission (initial oxygenation strategy)
Hilbert et al. [5]	2001	France	Randomised controlled trial	ICU	De novo hypoxemia	26	Mixed	NIV vs oxygen	Not specified	First line; face mask; within hours of ICU admission
Lemiale et al. [6]	2015	France and Belgium	Randomised controlled trial	ICU	De novo hypoxemia	191	Mixed	NIV Vs oxygen/HFNC	Excluded	191 patients randomised to immediate NIV as first-line support; ICU ventilator + face mask (majority), pressure-support mode; immediately post-randomisation
Lemiale et al. [7]	2015	Multicentre	Post hoc analysis of a prospective, multicentre cohort	ICU	De novo hypoxemia	142	Haematologic	NIV vs HFNC	Included	Initiated in 142/380 ; at ICU admission (first-line ICU strategy, assessed over first 48 hours)
Mokart et al. [10]	2020	Multinational	Prospective cohort	ICU	Ward/pre-ICU	202	Mixed	Neutropenia vs non-neutropenia	Not specified	Initiated in 202/1,481 patients; at ICU admission (initial oxygenation strategy)
Coudroy et al. [11]	2016	France	Retrospective cohort	ICU	De novo hypoxemia	55	Mixed	NIV vs HFNC	Excluded	Initiated in 55/115 (first-line); at ICU admission/within 6 hours of ARF onset (median 1 hour from ICU admission)
Lima et al. [12]	2021	Brazil	Retrospective cohort	ICU	De novo hypoxemia	226	Solid	Single-arm NIV cohort	Not specified	First line; mask
Rathi et al. [13]	2017	United States	Retrospective cohort	ICU	De novo hypoxemia	793	Mixed	NIV vs IMV	Not specified	First-line support in 793/1,614 patients; face mask; at ICU admission
Belenguer-Muncharaz et al. [14]	2013	Spain	Retrospective cohort	ICU/ward	ARDS	35	Haematologic	IMV vs NPPV	Not specified	Initiated in 35/41 (85%); first-line at ICU admission (CPAP sometimes used on ward pre-ICU)
Azoulay et al. [15]	2014	Multinational	Multicentre cohort	ICU	ARDS	387	Mixed	Descriptive ARDS cohort	Not specified	Initiated in 387/1,004; at ICU admission (first-line ventilatory strategy)
Türkoğlu et al. [16]	2013	Turkey	Semi-prospective cohort	Ward	ARDS	46	Haematologic	Descriptive ARDS cohort	Not specified	Initiated in 46/68; in ICU as first-line ARDS support with intubation on prespecified failure criteria
Grgić Medić et al. [17]	2015	Croatia	Prospective cohort	ICU	De novo hypoxemia	28	Haematologic	Descriptive ICU cohort	Included	Initiated in 28/170 patients with ARF; face mask or helmet system; at presentation with ARF, before intubation criteria were met
Siddiqui et al. [18]	2021	India	Prospective cohort	ICU	De novo hypoxemia	41	Haematologic	Descriptive ICU cohort	Excluded	Initiated in 41/101 patients; within 24 hours of ICU admission
Adda et al. [19]	2008	France	Retrospective cohort	ICU	De novo hypoxemia	99	Haematologic	Single-arm NIV cohort	Excluded	Median 0 days (IQR: 0-1) from ICU admission
Ferreira et al. [20]	2015	Brazil	Retrospective cohort	ICU	De novo hypoxemia	114	Mixed	Single-arm NIV cohort	Included	First line; oro-facial or total face mask; at ICU admission
Saillard et al. [21]	2020	France	Retrospective cohort	ICU	Cardiac dysfunction	63	Mixed	NIV vs HFNC	Not specified	NIV used as initial ventilation strategy
Meert et al. [22]	2011	Belgium	Retrospective cohort	ICU	De novo hypoxemia	41	Mixed	NIV vs IMV	Excluded	Initiated in 41/164 (25%); first-line trial in ICU prior to IMV
Squadrone et al. [23]	2010	Italy	Randomised clinical trial	ICU/ward	Ward/pre-ICU	10	Haematologic	CPAP vs oxygen	Excluded	Initiated in 10/40; rescue NIV at ICU entry after ward CPAP/oxygen failure (ICU admission on prespecified criteria)
Wermke et al. [24]	2012	Germany	Randomised controlled trial	Ward	Ward/pre-ICU	42	Haematologic	Intermittent NIV vs oxygen	Included	Early intermittent NIV delivered on the ward in arm B; full face mask; immediately after randomisation
Alptekinoglu Mendil et al. [25]	2021	Turkey	Randomised controlled trial	Ward	De novo hypoxemia	34	Haematologic	Context only: no NIV arm	Not specified	Initiated in 34/100 patients; after ward treatment failure
Lemiale et al. [26]	2014	France 16 medical	Multicentre post hoc cohort analysis of a previous RCT dataset	Ward	De novo hypoxemia	81	Mixed	NIV vs oxygen vs IMV; NIV success vs failure	Excluded	First-line face mask; at ICU admission
Barreto et al. [27]	2020	Brazil	Prospective cohort	ICU	De novo hypoxemia	59	Haematologic	Single-arm NIV cohort	Not specified	Initiated in 59/82 patients as first-line support; facial or nasal mask; immediately at ICU admission (within first 24 hours)
Gristina et al. [28]	2011	Italy	Retrospective analysis of a prospectively collected	ICU	De novo hypoxemia	274	Haematologic	NIV vs IMV	Not specified	Initiated in 274/1,302; at ICU admission (first-line; continuous for first 24 hours)

Early NIV vs Oxygen or HFNC in de novo Hypoxemic ARF

Pre-HFNC single-centre RCT (Hilbert et al., 2001; n = 52) showed lower intubation (46% vs 77%) and hospital mortality (50% vs 81%) with NIV [5]. In contrast, the largest contemporary RCT (Lemiale et al., 2015; n = 374) showed no reduction in intubation (38% vs 45%) or 28-day mortality (24% vs 27%) with early NIV vs oxygen/HFNC [6]. Large, adjusted cohorts (Efraim 2017, Mokart et al. 2020) found no benefit of initial NIV [4,10], while Coudroy et al. (2016) reported higher adjusted odds of intubation (aOR ≈3.25) and 28-day mortality (aOR ≈3.70) with NIV vs HFNC [11]. Across studies, NIV failure was ~30%-55% and was associated with poor survival [12-14].

Summary: In de novo hypoxemic ARF, early NIV did not improve intubation or survival vs oxygen or HFNC, signals of harm appear when NIV is prolonged without predefined stop rules. An exception may be ARF with cardiac dysfunction [4,6,10,11].

NIV in Established Cancer-Related ARDS

NIV failure was frequent and portended worse outcomes. In Azoulay et al. (2014) (n ≈1,000), NIV attempted in 39% with 71% failure (mild 63%, moderate 69%, severe 79%), failure independently predicted higher in-hospital mortality [15]. A nationwide cohort (Bris et al, 2024) confirmed high mortality in haematological malignancy-associated acute respiratory distress syndrome (HM-ARDS), driven by shock, invasive fungal infection, and malignancy type rather than ventilatory mode [2]. Smaller cohorts were concordant in Türkoğlu et al. (2013; failure 78%) [16], Belenguer-Muncharaz et al. (2013; failure 40%, mortality ~70% in failures vs <30% in successes) [14], Grgić Medić et al. (2015; failure 61%) [17], Lima et al. (2021; failure 52%, mortality ~70% vs ~20%) [12], and Siddiqui et al. (2021; failure 53%) [18].

Summary: In cancer-related ARDS, NIV failure is common (~60%-80%) and strongly predicts death; use time-limited NIV with predefined stop rules and intubate early if no prompt improvement.

Independent Predictors of NIV Failure and Mortality

Across multivariable analyses, reproducible independent predictors of NIV failure included higher illness severity (Simplified Acute Physiology Score II (SAPS II)/Sequential Organ Failure Assessment (SOFA)), shock/vasopressors, and more severe hypoxemia (lower arterial oxygen partial pressure to inspired oxygen fraction ratio (PaO₂/FiO₂)) [3,15,17,19,20]. Undetermined ARF aetiology and invasive fungal infection were consistently linked to higher failure and/or mortality [2,3,12,21]. In ARDS cohorts, NIV failure itself independently predicted death; delayed intubation and nosocomial infection further worsened outcomes [2,12,15]. In ARF with cardiac dysfunction, initial NIV was associated with lower adjusted ICU mortality vs oxygen/HFNC only [21]. Study-level predictors and adjustment sets appear in Table 2.

**Table 2:** Independent predictors of non-invasive ventilation (NIV) failure in adults with cancer and acute respiratory failure Multivariable (adjusted) predictors of NIV failure (defined as intubation during the index ARF episode) extracted from included studies. For each predictor, the table reports: clinical definition/cut-off (if provided), scenario (de novo hypoxaemia, ARDS, ward/pre-ICU, cardiac dysfunction), effect estimate (aOR or aHR) with 95% CI, p-value, model covariates (abridged), and study identifier. Direction of effect is shown as “↑ risk” or “↓ risk.” Where studies reported only strata (e.g., PaO₂/FiO₂ thresholds), the most predictive cut-off is listed; if multiple models were presented, the primary adjusted model was used. Predictors appearing in ≥2 studies are flagged as “consistent.” Univariate findings were not included. Consistency reflects replication across ≥2 independent cohorts. SAPS II/SOFA/APACHE II = illness-severity scores. ARF = acute respiratory failure, ARDS = acute respiratory distress syndrome, aOR = adjusted odds ratio, aHR = adjusted hazard ratio, CI = confidence interval, DNI = do-not-intubate, HFNC = high-flow nasal cannula, HM = haematological malignancy, HSCT = haematopoietic stem-cell transplantation, ICU = intensive care unit, IMV = invasive mechanical ventilation, NIV = non-invasive ventilation, PaO₂/FiO₂ = arterial oxygen partial pressure to inspired oxygen fraction ratio, SOFA = Sequential Organ Failure Assessment, PJP = *Pneumocystis jirovecii* pneumonia, IPA = invasive pulmonary aspergillosis, PaCO₂ = arterial carbon dioxide partial pressure, RRT = renal replacement therapy, LODS = Logistic Organ Dysfunction System, P/F = PaO₂/FiO₂ ratio, ALI = acute lung injury, LR = likelihood ratio (test), HL = Hosmer–Lemeshow (goodness-of-fit test), APACHE II = Acute Physiology and Chronic Health Evaluation II.

Study	Scenario	Outcome modelled	Model	Independent predictors (direction)	Adjusted effect (metric, 95% CI)	Key covariates
Azoulay et al. (2017) [4]	De novo hypoxemia	NIV failure/need for intubation (during index ARF)	Cause-specific Cox (competing risk = death)	Higher SOFA (↑); PaO₂/FiO₂ < 300 (↑); *Pneumocystis jirovecii* pneumonia (↑); Invasive pulmonary aspergillosis (↑); Undetermined ARF aetiology (↑); Age per year (↓).	SOFA per point HR: 1.09 (1.06-1.13); PaO₂/FiO₂ < 300 HR: 1.47 (1.05-2.07); PJP HR: 2.11 (1.42-3.14); IPA HR: 1.85 (1.21-2.85); Undetermined aetiology HR: 1.46 (1.09-1.98); Age per year HR: 0.92 (0.86-0.99).	Age; day 1 SOFA; PaO₂/FiO₂ class; ARF aetiology; initial oxygenation strategy (HFNC/NIV).
Coudroy et al. (2016) [11]	De novo hypoxemia	NIV failure/intubation (overall cohort)	Multivariable logistic regression	SAPS II (↑); Vasopressors within 24 hours (↑); NIV as first-line (↑).	SAPS II OR: 1.04 (1.00-1.08); Vasopressors OR: 4.12 (1.32-12.84); First-line NIV OR: 3.25 (1.39-7.60).	Age; PaO₂/FiO₂; immunosuppression type; vasopressors; cause of ARF; PaCO₂; year of ICU admission (forced). DNI excluded.
Adda et al. (2008) [19]	De novo hypoxemia	NIV failure/need for intubation (index ARF)	Multivariable logistic regression	Respiratory rate under NIV (↑); Delay from ICU admission to first NIV (↑); Vasopressors (↑); Renal replacement therapy (↑); ARDS at time of NIV (↑).	RR under NIV OR: 1.18 per breath/min (1.05-1.33); Delay OR: 2.00 per day (1.02-3.94); Vasopressors OR: 6.50 (1.59-26.53); RRT OR: 18.31 (1.99-168.65); ARDS OR: 77.71 (6.88-878.38).	Variables with p < 0.10 entered; final model as above; Hosmer-Lemeshow p = 0.64.
Lemiale et al. (2014) [26]	De novo hypoxemia	NIV failure/intubation (during ICU stay)	Multivariable logistic regression with random centre effect	Higher oxygen requirement at ICU admission (↑); More lung quadrants with infiltrates (↑); Haemodynamic dysfunction (↑).	Oxygen requirement OR: 1.11 per L/min (1.04-1.19); Lung quadrants OR: 1.56 per quadrant (1.20-2.02); Haemodynamic dysfunction OR: 2.25 (1.13-4.48).	Centre random effect; organ dysfunctions (LODS domains); infection; imaging extent; admission O₂ flow.
Barreto et al. (2020) [27]	De novo hypoxemia	NIV failure/intubation (during index ARF)	Multivariable logistic regression	Higher SOFA (↑); Higher respiratory rate (↑); Sepsis on ICU admission (↑).	SOFA OR: 1.35 (1.12-2.10); Respiratory rate OR: 1.10 (1.00-1.22) per bpm; Sepsis OR: 16.9 (1.93-149.26).	Age; SOFA; respiratory rate; P/F ratio; lactate; vasopressors; infection status.
Gristina et al. (2011) [28]	De novo hypoxemia	NIV failure/need for intubation (index ARF)	Multivariable logistic regression	Higher SAPS II (↑); ALI/ARDS at admission (↑).	SAPS II OR: 2.01 (1.01-4.03); ALI/ARDS OR: 2.27 (1.35-3.82).	Variables with p < 0.1 entered; final model retained SAPS II and ALI/ARDS (good fit: LR, p = 0.0012; HL, p = 0.76; no collinearity).

Prognosis After NIV Failure

Across cohorts, invasive mechanical ventilation after NIV failure carried very poor outcomes. Meert et al. (2011) (n = 164) found prior NIV before invasive mechanical ventilation (IMV) was independently associated with lower odds of survival to discharge (aOR ≈0.30) [22]. Rathi et al. (2017) (n = 1,614) reported an ICU mortality of 71%/in-hospital mortality of 80% after NIV failure vs 28%/47% with NIV success [13]. Similar patterns occurred in Lima et al. (2021) and Belenguer-Muncharaz et al. (2013) [12,14].

Summary: NIV failure is a strong negative prognostic marker (mortality often >70%); close monitoring and timely intubation are critical.

Ward/Pre-ICU Preventive Use

Two small single-centre ward RCTs in neutropenic/post-haematopoietic stem cell transplant (HSCT) units evaluated early non-invasive support. Squadrone et al. (2010) (helmet CPAP vs oxygen; n = 40) reported reduced ICU admission, intubation, and in-hospital mortality [23]. Wermke et al. (2012) (intermittent NIV + oxygen vs oxygen; n = 86) showed non-significant trends toward benefit [24].

Summary: Ward-based CPAP/NIV may avert deterioration in selected haematology patients, but evidence is limited (small, single-centre; largely pre-HFNC).

Summary of Results

Table 3 summarises the outcomes for scenarios 1, 2, and 5.

**Table 3:** Scenario-level outcomes for non-invasive ventilation (NIV) in adults with cancer and acute respiratory failure Summary of outcomes across four clinical contexts: (1) de novo hypoxaemia (not due to COPD or acute cardiogenic pulmonary oedema), (2) established ARDS, (3) ward/pre-ICU use in haematology settings, and (4) ARF with cardiac dysfunction. For each scenario, the table reports the number of contributing studies, approximate combined sample size, typical NIV failure (intubation) range, mortality range (primary time point in-hospital unless stated), main comparators (oxygen/HFNC/IMV), key qualitative signal (benefit/neutral/harm), and certainty rating (very low/low/moderate) based on study design, consistency, and precision. Where datasets may overlap, totals are approximate. Failure = intubation during the index ARF episode. ARF = acute respiratory failure, ARDS = acute respiratory distress syndrome, HFNC = high-flow nasal cannula, IMV = invasive mechanical ventilation, ICU = intensive care unit, HM = haematological malignancy, HSCT = haematopoietic stem-cell transplantation, RCT: randomised controlled trial, COPD = chronic obstructive pulmonary disease.

Scenario	Studies (n)	Patients on NIV (approx)	Median NIV failure % (IQR)	Median in-hospital mortality % (IQR)	Signal vs oxygen/HFNC	Notes
De novo hypoxemia	17	6,915	42.6% (38.0-52.8)	45.6% (42.2-54.5)	Neutral/harm vs HFNC	Largest RCT neutral; adjusted cohorts neutral/harm; use stop rules
Cardiac dysfunction	1	127	—	57.0% (57.0-57.0)	Favourable for NIV	Adjusted lower ICU mortality with initial NIV
ARDS	5	1,509	78.1% (62.0-84.0)	66.8% (59.5-72.1)	Harm (failure common)	Failure ≈60%-80%; early intubation if no rapid improvement
Ward/pre-ICU	3	226	60.0% (42.5-77.5)	15.0% (15.0-15.0)	Possible benefit (helmet CPAP)	Small single-centre, largely pre-HFNC; neutropenic/post-HSCT

Risk of Bias Within Studies

The four RCTs were open-label: two were small, single-centre ward trials, yielding “some concerns” for deviations from intended interventions. Most cohorts were at moderate risk of bias on the Newcastle-Ottawa Scale [9] due to confounding/selection. Risk of bias for observational studies is summarised in Table 4. Most cohorts were moderate risk due to residual confounding/selection, with a smaller subset at high risk (non-consecutive inclusion and/or substantial do-not-intubate (DNI) proportions). A minority achieved low-risk ratings with robust adjustment and complete follow-up.

**Table 4:** Risk of bias in observational cohorts assessed with the Newcastle-Ottawa Scale (NOS) This table reports NOS star ratings for the 22 observational cohorts across the three domains: Selection (max 4★), Comparability (max 2★), and Outcome (max 3★). Total score is out of 9★. Higher scores indicate lower risk of bias. Selection: representativeness of the exposed cohort, selection of the non-exposed cohort, ascertainment of exposure; outcome not present at start. Comparability: control for the most important confounders (e.g., ARF aetiology/ARDS severity, illness-severity scores, DNI status) and additional confounders. Outcome: assessment method; adequacy and length of follow-up; completeness of follow-up. Cohorts were qualitatively grouped as low risk (7-9★), moderate risk (5-6★), or high risk (≤4★) to aid interpretation. DNI = do-not-intubate, ARDS = acute respiratory distress syndrome, HM = haematologic malignancy, ARF = acute respiratory failure, NIV = non-invasive ventilation, ICU = intensive care unit, IMV = invasive mechanical ventilation, RCT: randomised controlled trial, ROB = risk of bias, PS = propensity score, IPW = inverse probability weighting, HLM = haematolymphoid malignancy.

NOS studies	NOS domain ratings			Overall summary		
Study details	Selection	Comparability	Outcome	Total	ROB	Justification
Liu et al., 2017 [1]	***	*	**	6/9 Stars	Moderate	Single-centre retrospective cohort. Clinician-set intubation thresholds. Incomplete adjustment for shock and oxygenation leaves confounding by indication.
Bris et al., 2024 [2]	****	**	**	8/9 Stars	Low	Large national cohort with mixed-effects adjustment. Limitations: coding-defined ARDS and no out-of-hospital death capture.
Contejean et al., 2016 [3]	****	**	***	9/9 Stars	Low	Large prospective multicentre HM ICU ARF cohort with blinded aetiology adjudication and comprehensive adjustment. Hospital mortality objectively ascertained.
Azoulay et al., 2017 [4]	****	**	***	9/9 Stars	Low	Prospective multinational cohort with excellent selection, strong adjustment including PS matching, and objective outcome ascertainment.
Lemiale et al., 2015 [7]	****	**	***	9/9 Stars	Low	Prospective multicentre cohort with excellent selection, strong propensity/IPW adjustment and objective outcomes, well suited to compare initial NIV vs oxygen.
Mokart et al., 2020 [10]	****	**	***	9/9 Stars	Low	Prospective multicentre cohort with excellent selection, strong confounding control using mixed models and propensity matching, and objective hospital mortality ascertainment.
Coudroy et al., 2016 [11]	***	**	***	8/9 Stars	Low	Single-centre retrospective ICU cohort with objective outcomes and reasonable adjustment for severity, oxygenation and vasopressors, limiting generalisability.
Lima et al., 2021 [12]	***	**	***	8/9 Stars	Low	Well-defined solid-tumour NIV cohort with robust adjustment for shock, oxygenation, and metabolic severity; objective outcomes minimize measurement bias despite single-centre, retrospective design. Well-defined solid-tumour NIV cohort with robust adjustment for shock, oxygenation, and metabolic severity; objective outcomes minimize measurement bias despite single-centre, retrospective design.
Rathi et al., 2017 [13]	***	**	***	8/9 Stars	Low	Large consecutive single-centre ICU cohort with objective outcomes and robust severity adjustment. Some residual confounding by indication and aetiology remains.
Belenguer-Muncharaz et al., 2013 [14]	***	*	***	7/9 Stars	Moderate	Solid outcome ascertainment in a well-defined cohort, but between-group confounding for NIV vs IMV is unadjusted and the single-centre retrospective design limits generalisability.
Azoulay et al., 2014 [15]	****	**	***	9/9 Stars	Low	Rigorous multicentre cancer-ARDS cohort using Berlin criteria with strong multivariable control, including ventilation strategy and invasive fungal infection, and objective mortality ascertainment.
Türkoğlu et al., 2013 [16]	***	*	***	7/9 Stars	Moderate	Clear single-centre HM-ARDS cohort with objective outcomes, but limited adjustment leaves residual confounding despite multivariable analysis.
Grgić Medić et al., 2015 [17]	***	**	***	8/9 Stars	Low	Prospective single-centre HM ICU cohort with objective outcomes and robust adjustment for severity and organ failure. Limitations include single centre design and residual confounding.
Siddiqui et al., 2021 [18]	***	**	***	8/9 Stars	Low	Prospective consecutive single-centre HLM cohort with objective outcomes and adjustment for severity and organ support. Generalisability limited by single centre.
Adda et al., 2008 [19]	***	*	**	6/9 Stars	Moderate	Single-centre retrospective cohort with objective outcomes. Limited adjustment for baseline severity and oxygenation leaves confounding by indication.
Ferreira et al., 2015 [20]	***	**	***	8/9 Stars	Low	Consecutive ICU NIV cohort with objective outcomes and reasonable adjustment for severity and aetiology. Single-centre design and residual confounding remain limitations.
Saillard et al., 2020 [21]	***	**	***	8/9 Stars	Low	Well-defined cohort with robust adjustment (severity, organ failure, aetiology) and propensity matching. Objective outcomes minimise measurement bias despite single-centre design.
Meert et al., 2011 [22]	***	*	***	7/9 Stars	Moderate	Clear single-centre IMV cohort with objective outcomes. Modest adjustment without key severity or oxygenation covariates leaves confounding by indication.
Alptekinoğlu Mendil et al., 2021 [25]	***	**	***	8/9 Stars	Low	Open label ward RCT. Concealment plausible but sequence details sparse. Minor post-randomisation exclusions. Objective outcomes limit measurement bias.
Lemiale et al., 2014 [26]	***	**	***	8/9 Stars	Low	Prospective multicentre ICU cohort with prespecified intubation criteria and robust multivariable modelling, including centre effects. Excluding baseline DNI introduces minor selection bias.
Barreto et al., 2020 [27]	***	**	***	8/9 Stars	Low	Prospective protocolised single-centre cohort with objective outcomes and solid adjustment, limited by single-centre design.
Gristina et al., 2011 [28]	****	**	***	9/9 Stars	Low	Exemplary multicentre cohort with rigorous selection and strong propensity-adjusted comparability, using objective outcomes. Well suited to inform initial NIV versus IMV strategies in HM ARF.

Risk of bias for randomised trials is presented in Table 5. Most trials had some concerns, primarily for deviations from intended interventions in open-label designs, while randomisation, outcome measurement, and missing data were generally low risk.

**Table 5:** Risk of bias in randomised trials assessed with RoB 2 This table summarises domain-level and overall risk-of-bias judgements for the four randomised trials using the Cochrane Risk of Bias 2 tool [8]. Domains: (1) randomisation process, (2) deviations from intended interventions, (3) missing outcome data, (4) measurement of the outcome, and (5) selection of the reported result. Judgements are Low risk, Some concerns, or High risk, with the overall rating following RoB 2 guidance [8] (worst-domain governs overall where applicable). RoB 2 = Cochrane Risk of Bias 2, RCT = randomised controlled trial, HFNC = high-flow nasal cannula, SAP = statistical analysis plan.

Study details			ROB 2 domains			Overall risk of bias	
Study	Randomisation process	Deviations from intended interventions	Missing outcome data	Measurement of outcomes	Selection of the reported result	Judgment	Reasons
Hilbert et al., 2001 [5]	Some concerns	Some concerns	Low risk	Low risk	Some concerns	Some concerns	Small, single-centre, open-label trial with limited sequence details, objective outcomes, and predefined intubation criteria mitigate.
Lemiale et al., 2015 [6]	Low risk	Some concerns	Low risk	Low risk	Low risk	Some concerns	Robust randomisation and prespecified SAP, but open-label design with allowable co-interventions (notably HFNC imbalance) introduces potential performance bias.
Squadrone et al., 2010 [23]	Low risk	Some concerns	Low risk	Low risk	Some concerns	Some concerns	Strong randomisation and objective outcomes, but open-label delivery with potential co-intervention/monitoring differences and lack of a public protocol introduce performance/reporting concerns.
Wermke et al., 2012 [24]	Some concerns	High Risk	Low risk	Low risk	Some concerns	High risk	The mandated cross-over and open-label design introduce substantial performance/deviation bias despite objective outcome measurement and low missing data.

Discussion

Modern evidence does not support routine NIV for every cancer-related ARF. The early single-centre RCT signal [5] has not been replicated in the HFNC era. Contemporary trials and adjusted cohorts show no dependable benefit of NIV over oxygen or HFNC in de novo hypoxemia and may suggest harm when NIV is prolonged in non-responders or used without clear stop rules [4,6,10,11]. In cancer-related ARDS, NIV fails in about 60%-80% of cases and failure strongly predicts death. High-risk features include shock, low PaO₂/FiO₂, invasive fungal infection, higher severity scores, and an undetermined cause of ARF [2,3,12,15,21]. Two ward trials suggested a niche for early helmet CPAP/NIV in selected neutropenic or post-HSCT patients [23,24]. Across settings, NIV failure persistently signals exceedingly higher rates of mortality [12-14,22,25-27].

In short, our findings do not argue against NIV as a modality, but against indiscriminate or prolonged NIV in high-risk de novo hypoxemic ARF. The NIV that looked helpful before HFNC and standardised pathways is now often found to be neutral and may be associated with worse outcomes when NIV delays needed intubation. Where HFNC is limited or unavailable, a brief, protocol driven NIV trial can be reasonable if strict stop rules and rapid access to intubation are applied.

Limitations

No meta-analysis was performed, as the studies were too heterogeneous. The certainty of our results is limited by the mainly observational designs, confounding by indication, variable handling of do-not-intubate (DNI) orders, and differences in NIV protocols, interfaces, and intubation thresholds. Some large cohorts may overlap, so the pooled number of patients is approximate. The included studies were also limited to the English language, so publication and language bias are possible. These issues were reduced by implementing a focus on adjusted analyses and a scenario-based qualitative synthesis; however, they cannot be fully eliminated.

Practice Signals

In de novo hypoxemia, HFNC is preferred. If NIV is trialled, set strict stop rules and reassess at 60-90 minutes. Intubate early if no response [6,11]. In ARDS, do not persist with NIV without rapid improvement. Intubate early [2,15]. In ARF with cardiac dysfunction, NIV may be favourable [21]. For ward treatment (neutropenic/post-HSCT), consider CPAP/NIV only with trained staff, continuous monitoring, and fast ICU access [10,24,25,28].

Pragmatic Stop Rules (60-90 Minutes)

Target peripheral capillary oxygen saturation (SpO₂) ≥92%, respiratory rate ↓ ≥20%, PaO₂/FiO₂ ↑ ≥20%, and no rising work of breathing or new haemodynamic or neurological instability. If targets are not met, or the patient deteriorates, intubate. Do not escalate NIV further.

Harms and Complications

Device complications were uncommon, but delayed intubation after NIV failure was linked to more nosocomial infection and higher mortality. It is advisable to avoid prolonged NIV in non-responders.

Research Priorities

There is a need for further stratified RCTs of HFNC vs NIV (helmet vs mask) with protocolised stop rules and oncology-specific subgroup, ward implementation trials focused on staffing, monitoring, and escalation, and standardised reporting of intubation criteria, DNI status, and ARF aetiology.

## Conclusions

In adults with cancer and ARF, NIV’s benefit is scenario dependent. For de novo hypoxemia, NIV offers no clear advantage over oxygen/HFNC and may be harmful if prolonged in non-responders. In ARDS, NIV often fails and failure strongly predicts mortality. A targeted use of NIV remains beneficial for cardiac dysfunction and selected ward populations. When NIV is attempted, tight monitoring and early intubation for non-responders are essential to avoid harm.

## Appendices

Appendix 1a

PubMed (MEDLINE) Search run: 31 August 2025 Limits: English language, no date restriction

Search strategy:

("non-invasive ventilation"[Title/Abstract] OR "NIV"[Title/Abstract] OR "noninvasive ventilation"[Title/Abstract]) AND ("cancer"[Title/Abstract] OR "malignancy"[Title/Abstract] OR "oncology"[Title/Abstract] OR "hematologic malignancy"[Title/Abstract]) AND ("acute respiratory failure"[Title/Abstract] OR "respiratory failure"[Title/Abstract] OR "ARDS"[Title/Abstract])

Appendix 1b

Scopus Search run: 31 August 2025 Limits: Articles and reviews; publication year 2010-2025; English language

Search strategy:

TITLE-ABS-KEY("non-invasive ventilation" OR "NIV" OR "noninvasive ventilation" OR "CPAP" OR "BiPAP") AND TITLE-ABS-KEY("acute respiratory failure" OR "respiratory failure" OR "ARDS") AND TITLE-ABS-KEY("hematologic malignancy" OR "solid tumor" OR "leukemia" OR "lymphoma") AND TITLE-ABS-KEY("mortality" OR "intubation" OR "outcome" OR "length of stay" OR "NIV failure") AND TITLE-ABS-KEY("ICU" OR "intensive care" OR "critical care")

Appendix 1c

Cochrane CENTRAL Search run: 31 August 2025 Limits: Studies published between 2010 and 2025; English language

Search strategy:

(non-invasive ventilation OR NIV OR noninvasive ventilation OR CPAP OR BiPAP) AND (cancer OR malignancy OR neoplasm OR oncology OR hematologic malignancy OR leukemia OR lymphoma OR solid tumor) AND (acute respiratory failure OR respiratory failure OR ARDS)
